# Upregulation of DDAH2 Limits Pulmonary Hypertension and Right Ventricular Hypertrophy During Chronic Hypoxia in *Ddah1* Knockout Mice

**DOI:** 10.3389/fphys.2020.597559

**Published:** 2020-11-12

**Authors:** Juliane Hannemann, Antonia Glatzel, Jonas Hillig, Julia Zummack, Udo Schumacher, Nicole Lüneburg, Lars Harbaum, Rainer Böger

**Affiliations:** ^1^Institute of Clinical Pharmacology and Toxicology, University Medical Center Hamburg-Eppendorf, Hamburg, Germany; ^2^Institute DECIPHER (German-Chilean Institute for Research on Pulmonary Hypoxia and Its Health Sequelae), Hamburg, Germany; ^3^Institute of Anatomy and Experimental Morphology, University Medical Center Hamburg-Eppendorf, Hamburg, Germany; ^4^Department of Pulmonology, II. Medical Clinic and Policlinic, University Medical Center Hamburg-Eppendorf, Hamburg, Germany

**Keywords:** hypoxia, nitric oxide, hypoxic vasoconstriction, endothelium, ADMA, DDAH

## Abstract

**Objective:** Chronic hypoxia causes pulmonary vasoconstriction leading to pulmonary hypertension and right ventricular hypertrophy. Asymmetric dimethylarginine (ADMA) is an endogenous inhibitor of nitric oxide (NO) synthesis; its level increases in hypoxia (HX) concomitantly with reduced activity of dimethylarginine dimethylaminohydrolases (DDAH-1 and DDAH-2), enzymes metabolizing ADMA. Ddah1 knockout (KO) mice may therefore help to understand the pathophysiological roles of this enzyme and its substrate, ADMA, in the development of hypoxia-associated pulmonary hypertension.

**Methods:** Ddah1 KO mice and their wild-type (WT) littermates were subjected to normoxia (NX) or for 21 days. We measured ADMA concentration in plasma and lungs, DDAH1 and DDAH2 mRNA and protein expression in the lungs, right ventricular systolic pressure (RVSP), right ventricular hypertrophy by the Fulton index, and cardiomyocyte hypertrophy by dystrophin staining of the heart.

**Results:** Ddah1 KO mice had higher ADMA concentrations in plasma and in lung tissue than WT in NX (*p* < 0.05). ADMA significantly increased in WT-HX in plasma and lungs, while there were no significant differences in WT-HX vs. KO-HX. This finding was paralleled by a 38 ± 13% reduction in Ddah1 but not Ddah2 mRNA expression, and reduced DDAH1 protein expression but stable DDAH2 protein levels in WT mice. Ddah1 KO mice showed significant elevation of DDAH2 protein but not mRNA levels, which further increased in HX. HX led to increased RVSP and right ventricular hypertrophy in both, WT and KO mice, with no significant differences between both genotypes.

**Conclusions:** Chronic hypoxia causes an elevation of ADMA, which may impair NO production and lead to endothelial dysfunction and vasoconstriction. Downregulation of DDAH1 expression and activity may be involved in this; however, knockout of the Ddah1 gene does not modify the hypoxia-induced pathophysiological changes of pulmonary blood pressure and right ventricular hypertrophy, possibly due to compensatory upregulation of DDAH2 protein.

## Introduction

Pulmonary arterial hypertension is a severe disease that develops either primarily or because of pathological conditions such as chronic hypoxia (HX; Humbert et al., 2004). We have recently reported that the prevalence of pulmonary arterial pressures above 30 mm Hg is as high as 9.2% in human subjects that were chronically exposed to altitudes above 3,500 m (Brito et al., 2018). Individuals living permanently at high altitude show a similarly high prevalence of high altitude-pulmonary hypertension (Penaloza and Arias-Stella, 2007). HX leads to vasoconstriction of vascular smooth muscle cells, resulting in hypertrophy of pulmonary arterioles, which form a characteristic histological feature of pulmonary arterial hypertension. Elevated pulmonary arterial pressure in turn results in right ventricular hypertrophy and failure (Grimminger et al., 2017).

The vascular endothelium plays a pivotal role in regulating systemic and pulmonary vascular tone, mainly by secreting nitric oxide (NO), a major mediator of vasodilation (Moncada and Higgs, 1993). Its formation in the vascular endothelium by endothelial NO synthase (eNOS) is regulated, besides other mechanisms, through competitive inhibition by asymmetric dimethylarginine (ADMA; Böger, 2003). Elevated plasma concentrations of ADMA are associated with endothelial dysfunction (Böger et al., 1998), cardiovascular disease (Böger et al., 2009a), and are predictive for mortality in the general population (Böger et al., 2009b). Both, synthesis of ADMA through arginine methylation of proteins (for Review, see Bulau et al., 2007) and its metabolic degradation by dimethylarginine dimethylaminohydrolases (DDAH), are present in the lung (Leiper et al., 1999). Specifically, Millatt and co-workers reported that DDAH1 protein expression was reduced in the lungs of rats exposed to chronic hypoxia (Millatt et al., 2003), and our group reported that DDAH activity in the lungs of rats exposed to chronic hypoxia was reduced concomitantly with increased ADMA (Lüneburg et al., 2016). By contrast, DDAH1 overexpression decreased acute hypoxic pulmonary vasoconstriction, but it did not change chronic hypoxia-induced pulmonary hypertension in mice (Bakr et al., 2013). Furthermore, heterozygous knockout (KO) of Ddah1 in mice resulted in impaired NO-dependent vasodilation of pulmonary arteries (Leiper et al., 2007). These effects were reversible by L-arginine, which is consistent with the competitive nature of eNOS by ADMA.

Our groups recently described a global Ddah1 KO mouse (Hu et al., 2011). This mouse model is characterized by elevated ADMA levels, virtually absent DDAH activity in all organs tested, and an about 20 mm Hg higher systemic arterial blood pressure. In the present study, we investigated whether chronic exposure to hypoxia results in changes of DDAH expression, ADMA concentration, pulmonary arterial pressure, and right ventricular hypertrophy in mice, and whether Ddah1 knockout affects these responses to hypoxia.

## Materials and Methods

### Animals and Hypoxia Study Protocol

The global-Ddah1 knockout mice used in the present study were described previously (Hu et al., 2011). Heterozygous crossbreeding was used to generate Ddah1^−^/^−^, Ddah1^+^/^−^, and wild-type (WT) mice, the latter being used as controls. Male and female mice were included in the study at the age of 9 weeks and were maintained for 4 weeks under normoxic [21% oxygen, normoxia (NX) groups] or hypoxic conditions (10 ± 1% oxygen, HX groups). The chamber environment was monitored using an oxygen analyzer. All animals had free access to food and water and were housed under controlled conditions of 20 ± 2°C, 45–65% humidity, and a light-dark cycle of 12:12 h. Body weight was measured on days 0, 7, 14, 21, and 28. Blood samples were taken on days 0 and 28. The use of animals was consistent with the Guide for the Care and Use of Laboratory Animals published by the US National Institutes of Health (NIH publication No. 85–23, revised 1996), and the experimental protocol was approved by the local authority (Amt für Gesundheit und Verbraucherschutz Hamburg, approval no. 02/16).

At the end of the study protocol (day 28), mice were anesthetized with 1.6 g/kg of urethane and ventilated by endotracheal intubation using a Minivent mouse ventilator type 845 (Hugo Sachs Elektronik, Hugstetten, Germany; 200 breaths/min). Right ventricular hemodynamics were measured after preparation of the jugular vein and insertion of a 1.2 F pressure catheter connected to an advantage pressure volume system (Transonic Systems Inc., Ithaca, NY, United States), which was advanced into the right ventricle (RV). Mean right ventricular systolic pressure (RVSP) was calculated using the Iox 2.9.5.68 software (Emka Technologies, Paris, France). After completion of the hemodynamic measurements, mice were euthanized by cervical dislocation, exsanguinated, and the heart and lungs were collected. Heart and lungs were flushed with PBS to remove all remaining blood. The right lung was immediately frozen in liquid nitrogen and stored at −80°C for RNA extraction. The left lung was inflated through the trachea and fixed in 4% formalin for histological analysis. The RV free wall, left ventricle (LV) and septum tissue were harvested and weighted. RV hypertrophy was calculated by Fulton index as the weight ratio of RV and (LV + septum). Then, RV tissue was saved frozen (−80°C) for biochemical analyses. Hematocrit was obtained for each mouse immediately after euthanasia by using a Hematocrit Centrifuge (Hettich, Tuttlingen, Germany).

### Measurement of ADMA and Symmetric Dimethylarginine in Plasma and Lung Tissue by Liquid Chromatography–Tandem Mass Spectrometry

Plasma samples were centrifuged immediately and stored at −20°C until analysis. Frozen mouse lung tissue was homogenized by grinding and repeated snap freezing in liquid nitrogen, and resuspended in 60 μl of PBS. Dimethylarginine concentrations were determined by liquid chromatography–tandem mass spectrometry (LC-MS/MS) using a previously validated method (Schwedhelm et al., 2007). In brief, 25 μl of plasma were diluted with stable isotope labeled internal standards. Proteins were precipitated with methanol, the guanidine compounds were converted to their butyl esters and analyzed by LC–MS/MS (Varian 1,200 MS, Agilent Technologies, Santa Clara, United States). Quantification was performed by calculation of peak area ratios and calibration with known concentrations of ADMA in dialyzed EDTA plasma. The analytical range of the method was validated from 0.05 to 4 μmol/L, and mean coefficients of variation were ≤5%.

### Analysis of Ddah1 and Ddah2 mRNA Expression

Total RNA was isolated from homogenized lung tissue using 1 ml of Trizol (ThermoFisher Scientific, Waltham, MA, United States) per sample. Sample clean-up and on-column removal of genomic DNA (gDNA) were performed using PureLink™ RNA Mini Kit in combination with PureLink™ DNase (both ThermoFisher). RNA integrity and complete gDNA digestion were verified by agarose gel electrophoresis; RNA samples were stored at −80°C until further use. About 2.5 μg total RNA were reverse-transcribed with SuperScript™ IV VILO™ (ThermoFisher). The reaction mix contained 2.5 μg total RNA, 4 μl SuperScript™ IV VILO™ Mastermix, and nuclease-free water to adjust the reaction volume to 20 μl; reverse transcription took place according to the following protocol: primer annealing: 10 min at 25°C; reverse transcription: 10 min at 50°C; and enzyme deactivation: 5 min at 85°C, cool-down to 4°C.

Quantitative real-time PCR (qRT-PCR) was performed in a total volume of 10 μl using 12.5 ng cDNA, Taqman Fast Advanced Master Mix, and gene-specific Taqman™ assays containing unlabeled gene-specific primers and 5'-FAM TaqMan™ MGB probe with 3'-nonfluorescent quencher (all ThermoFisher). The following assays were used: Ddah1 (Mm01319451_m1), Ddah2 (Mm00516768_m1), and Ppia (Mm02342430_g1), respectively. All samples were prepared as technical triplicates; non-template controls were included for each assay. The reaction was run on a Quantstudio 5 System (ThermoFisher) using the following cycling conditions: UNG incubation: 2 min at 50°C; and activation 10 min at 95°C, 40 cycles of denaturation (15 s at 95°C) and annealing/extension (1 min at 60°C). Relative gene expression was determined using the ∆∆Ct method after normalization against Ppia, which showed stable expression in all samples in prior experiments.

### Western Blot Analysis of DDAH1 and DDAH2 Protein

Frozen mouse lung tissue was homogenized by grinding and immediate transfer of the lung powder to 20 μl of T-PER tissue lysis buffer (Thermofisher, Waltham, Massachusetts, United States) containing 1x protease inhibitor (complete ULTRA, EDTA-free, Merck, Darmstadt, Germany) per milligram tissue according to the manufacturer’s instruction. Cell lysates were loaded onto a QIAshredder column (Qiagen, Hilden, Germany) and homogenized by centrifugation for 2 min at 16.100 × *g* and 4°C. Around 35 μg of samples were boiled in beta-mercaptoethanol containing sample buffer and subjected to 10% SDS-PAGE. Subsequently, proteins were transferred onto a 0.45 μm nitrocellulose membrane (GE Healthcare, Munich, Germany). Membranes were incubated with the primary antibodies over night at 4°C (anti-DDAH2: 1:1,000, Abcam ab184166; anti-DDAH1: 1:1,000, Invitrogen PA5-95366; anti-*β*-tubulin: 1:500, Abcam ab6046; Cambridge, United Kingdom) and the appropriate secondary antibody (A0545, Merck, Darmstadt, Germany), diluted 1:1,000 for DDAH2 and 1:10,000 for DDAH1 and β-tubulin, for 1 h at room temperature. Protein was detected using enhanced chemiluminescence detection according to the manufacturer’s protocol (GE Healthcare, Munich, Germany).

### Immunohistochemical Staining for DDAH2 in Lung Sections

For immunohistochemical detection of DDAH2, 5 μm lung sections were de-paraffinized and pre-treated with Dako Retrieval solution S2367 (pH 9, Dako, Hamburg, Germany) for 2 × 4 min in a microwave. The primary antibody against DDAH2 (Abcam ab232694; 10 μg/ml) was incubated on the slides for 60 min at room temperature. For antibody detection, the Dako REAL™ Detection System (K5005) was used according to the manufacturer’s instructions. Nuclei were counterstained using Mayer’s hemalum solution.

### Assessment of the Effects of Hypoxia on the Right Ventricle and Lung Circulatory System

For determining the wet weight of the heart, the right ventricle was carefully dissected from the LV and septum, and both parts of the heart were weighed separately. The relative weight of the right ventricle was expressed as [weight (right ventricle)/weight (left ventricle + septum)].

Histological analysis was performed using formalin-fixed, 5 μm thick paraffin-embedded heart and lung tissues. Cardiomyocyte hypertrophy was measured using cardiac sections that were immunostained using an anti-dystrophin antibody (Merck, Darmstadt, Germany). Mean cardiomyocyte cross-sectional area was separately determined for the right and left ventricles in digitized sections using the Panoramic Viewer software (3DHISTECH Ltd., Budapest, Hungary); Around 400 cardiomyocytes were analyzed per heart from at least four mice per group.

### Statistical Analyses

Statistical analysis of *in vivo* hemodynamics was performed using a one-way ANOVA with Dunnett’s test for exposure group (Normoxia vs. Hypoxia, WT vs. Ddah1^−^/^−^). Statistical analysis of differences in dimethylarginine concentrations in lung tissue was performed using two-sided Mann Whitney test. Expression of DDAH1 and DDAH2 mRNA and protein in lung tissues was tested for statistically significant differences using one-way ANOVA followed by Tukey’s multiple comparison test. Statistical analyses were performed using GraphPad Prism version 6.0 (GraphPad Software Inc. San Diego, CA, United States). All *p*-values were two sided and *p* < 0.05 was taken as statistically significant. Data are presented as mean ± SEM.

## Results

### Characterization of the Hypoxia Mouse Model

Wild-type and Ddah1^−^/^−^ mice that were kept under normoxic conditions showed a constant gain of body weight throughout the experimental period. During hypoxia, mice lost weight during the first week; thereafter, the increase in body weight was parallel to the normoxic groups (data not shown). There was no significant weight difference between male and female mice. In normoxia, hematocrit was 48.4 ± 4.4% in WT mice and 63.3 ± 2.9% in Ddah1^−^/^−^ mice (*p* < 0.05). Hypoxia caused a significant increase in hematocrit to 68.7 ± 4.9% (WT) and 84.3 ± 9.6% (Ddah1^−^/^−^), respectively (both *p* < 0.05 vs. normoxia; *p* = n.s. between genotypes).

### ADMA Plasma Concentrations in Normoxia and Hypoxia

**Figure 1:**
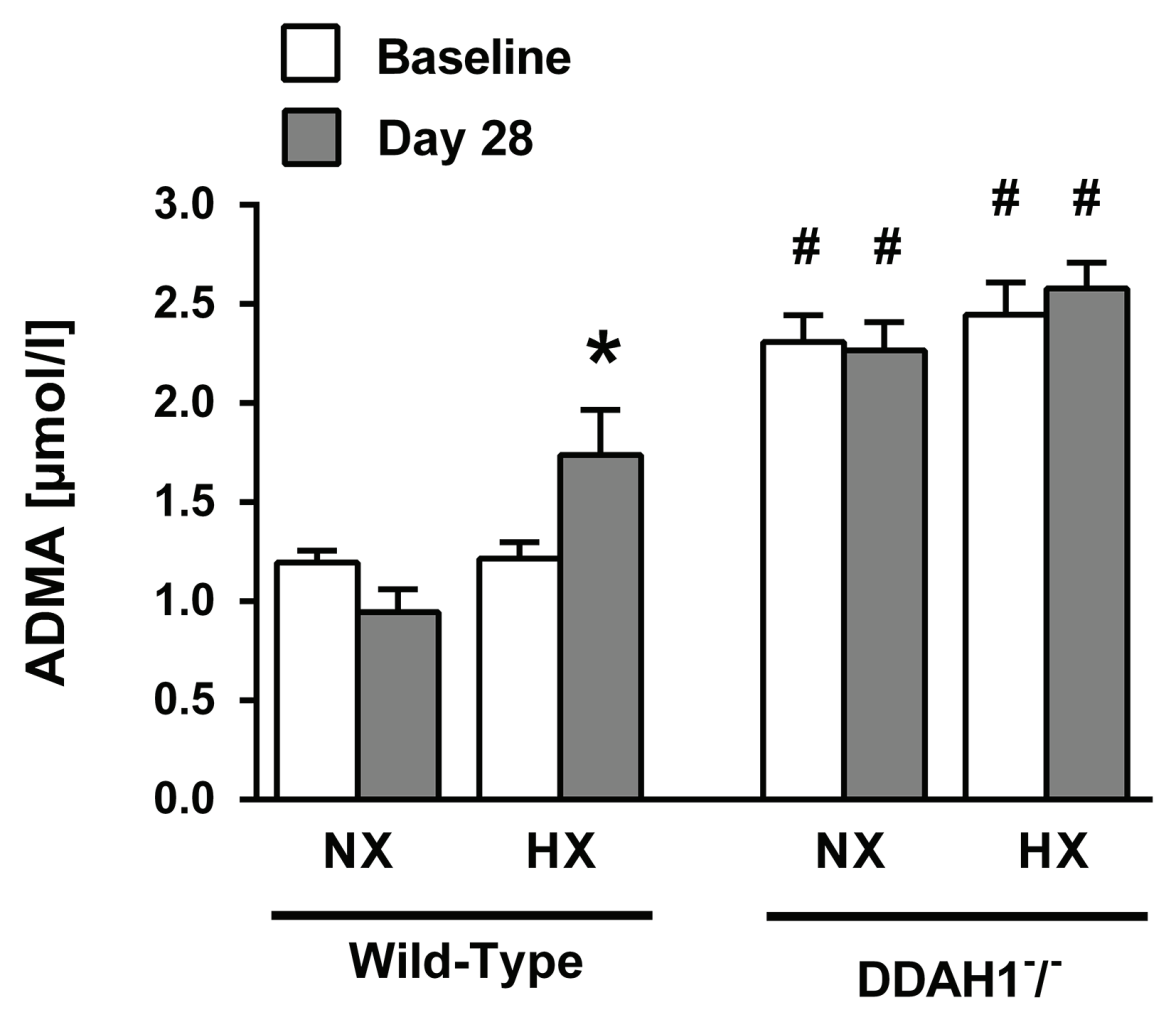
Asymmetric dimethylarginine (ADMA) plasma concentrations at the beginning of the experiment (baseline) and after 4 weeks of normoxia (NX) or hypoxia (HX; Day 28). Data are mean ± SEM of 8–12 animals per group. ^*^p < 0.05 vs. normoxia; ^#^*p* < 0.05 vs. wild-type (WT). NX, normoxia (21% O_2_); HX, hypoxia (10% O_2_).

In normoxia, ADMA plasma concentration was significantly higher in Ddah1^−^/^−^ than in WT mice (2.31 ± 0.33 μmol/l vs. 1.20 ± 0.17 μmol/l; *p* < 0.05). Hypoxia caused a significant increase in ADMA concentration in WT mice (1.74 ± 0.86 μmol/l; *p* < 0.05 vs. normoxia), but no further increase in Ddah1^−^/^−^ mice (2.58 ± 0.58 μmol/l; *p* = n.s. vs. normoxia) was observed (Figure 1).

**Figure 2:**
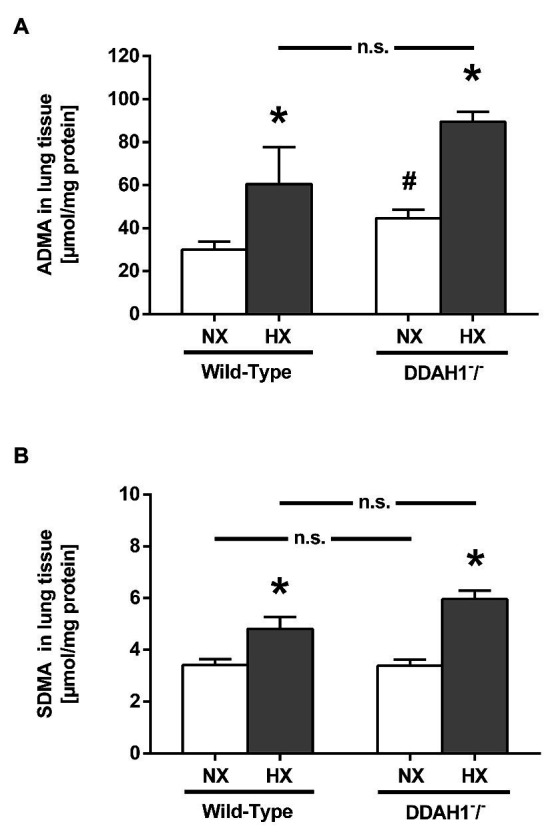
ADMA **(A)** and symmetric dimethylarginine (SDMA) concentrations **(B)** in lung tissue of mice after 4 weeks of NX or HX. Data are mean ± SEM of 3–7 animals per group. ^*^p < 0.05 vs. normoxia; ^#^*p* < 0.05 vs. WT. NX, normoxia (21% O_2_); HX, hypoxia (10% O_2_).

In lung tissue, both ADMA and SDMA concentrations were elevated in hypoxia as compared to normoxia, both in WT and in Ddah1^−^/^−^ mice (Figures 2A,B). ADMA was significantly elevated in lung tissue of Ddah1^−^/^−^ mice in normoxia as compared to WT littermates (44.6 ± 9.7 μmol/g protein vs. 30.0 ± 9.3 μmol/g protein; *p* < 0.05), while SDMA concentration remained unchanged (3.4 ± 0.6 μmol/g protein vs. 3.4 ± 0.6 μmol/g protein; *p* = n.s.). There was no significant difference in lung ADMA concentration between WT and Ddah1^−^/^−^ mice in hypoxia (Figure 2A).

### Expression of Ddah1 and Ddah2 mRNA in Normoxia and Hypoxia

**Figure 3:**
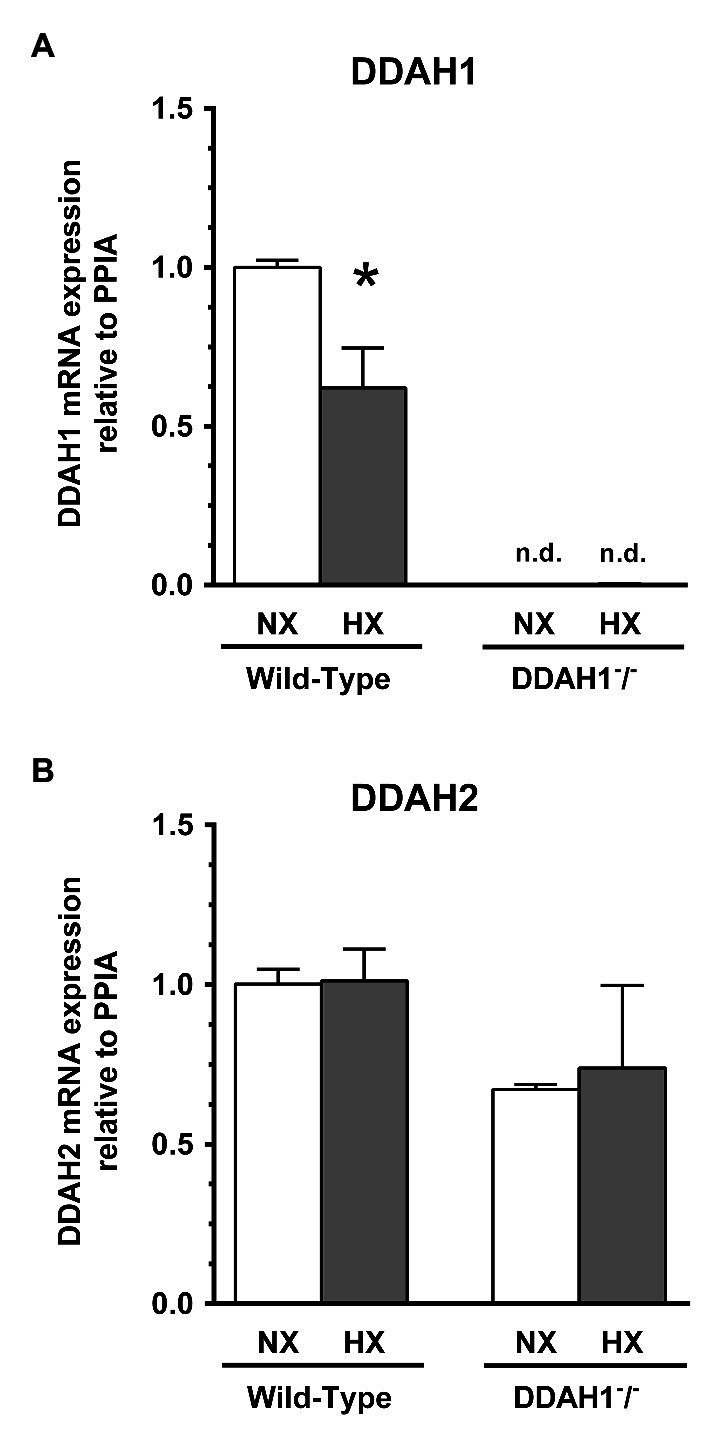
Expression of DDAH1 **(A)** and DDAH2 **(B)** mRNA in lung homogenates from wild-type and DDAH1^−^/^−^ mice kept under normoxia or hypoxia for 4 weeks. mRNA expression was normalized to cyclophilin A (peptidylprolyl isomerase A, PPIA) as housekeeping gene, and to wild-type mice under normoxia as control group. Data are mean ± SEM of 5–7 animals per group. ^*^p < 0.05 vs. normoxia; n.d., not detectable. NX, normoxia (21% O_2_); HX, hypoxia (10% O_2_).

Relative Ddah1 and Ddah2 mRNA expression was determined in lung tissues of Ddah1^−^/^−^ and WT mice under normoxic conditions. Ddah1 mRNA was almost completely absent in Ddah1^−^/^−^ mice whereas Ddah2 mRNA levels were not significantly different between genotypes (Figure 3).

Ddah1 mRNA expression in the lung was significantly lower by 37.9 ± 12.5% in hypoxia as compared to normoxia in WT mice (*p* < 0.05; Figure 3A); in Ddah1^−^/^−^ mice no relevant Ddah1 mRNA expression was detectable as expected. Ddah2 mRNA expression was not significantly different between normoxia and hypoxia in both groups (Figure 3B).

### Western Blot Analysis of DDAH1 and DDAH2 Protein Expression in Normoxia and Hypoxia

**Figure 4:**
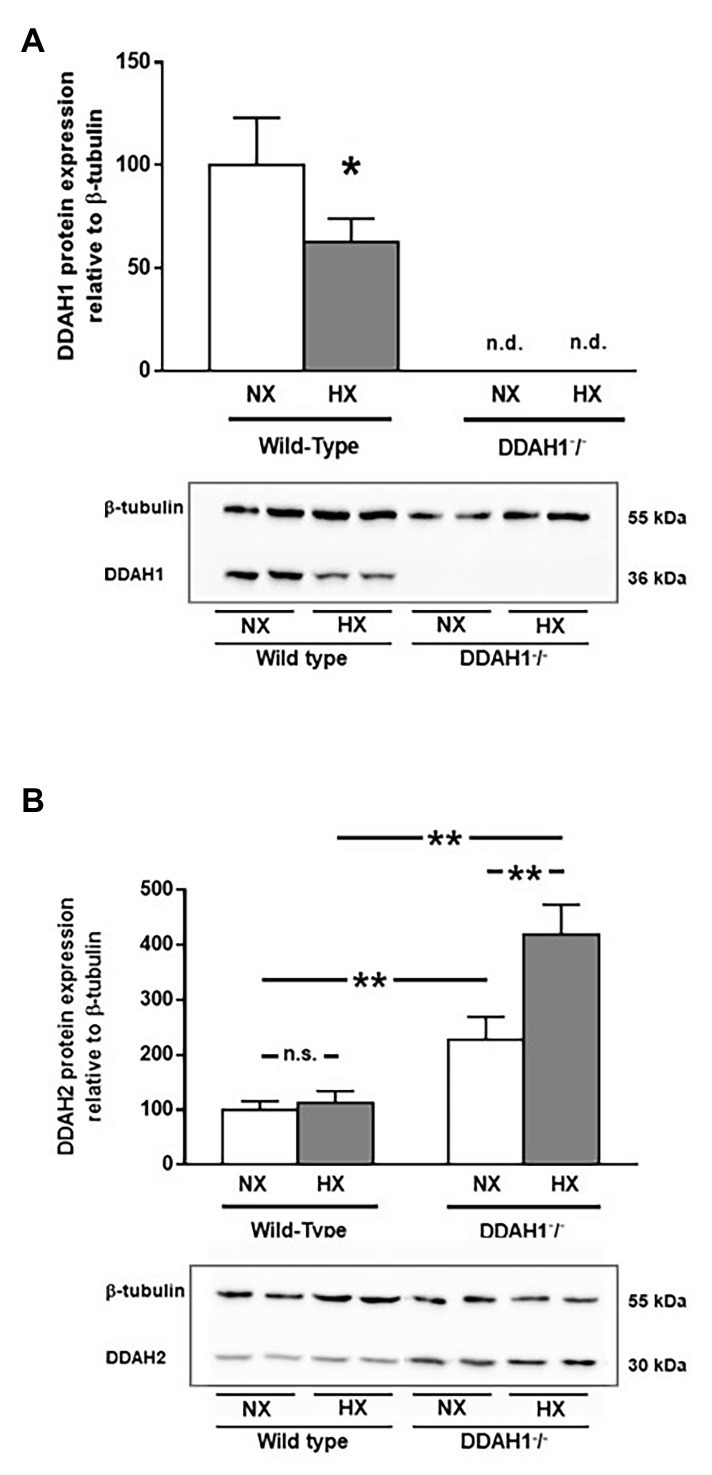
Western Blot analysis of DDAH1 **(A)** and DDAH2 **(B)** protein in lung homogenates from wild-type and DDAH1^−^/^−^ mice kept under normoxia or hypoxia for 4 weeks. Protein expression was normalized to ß-tubulin, and to wild-type mice under normoxia as control group. Data are mean ± SEM of 4–5 animals per group. ^*^p < 0.05; ^**^p < 0.0001 as indicated; n.s., not significant; n.d., not detectable. NX, normoxia (21% O_2_); HX, hypoxia (10% O_2_).

In WT controls, hypoxia caused a 37% reduction in DDAH1 protein in the lung (*p* = 0.036). DDAH1 protein levels were not detectable in Ddah1^−^/^−^ mice (Figure 4A).

DDAH2 protein levels were significantly higher in Ddah1^−^/^−^ mice than in WT controls. Hypoxia caused no significant change in DDAH2 protein expression in WT mice. In Ddah1^−^/^−^ mice, DDAH2 protein levels were upregulated by 2.3-fold in normoxia (*p* < 0.001), and it further increased in hypoxia to 4.2-fold over WT (*p* < 0.0001 vs. WT NX; *p* < 0.0001 vs. Ddah1^−^/^−^ NX; Figure 4B).

### Immunohistochemical Staining for DDAH2 in Lung Sections

**Figure 5:**
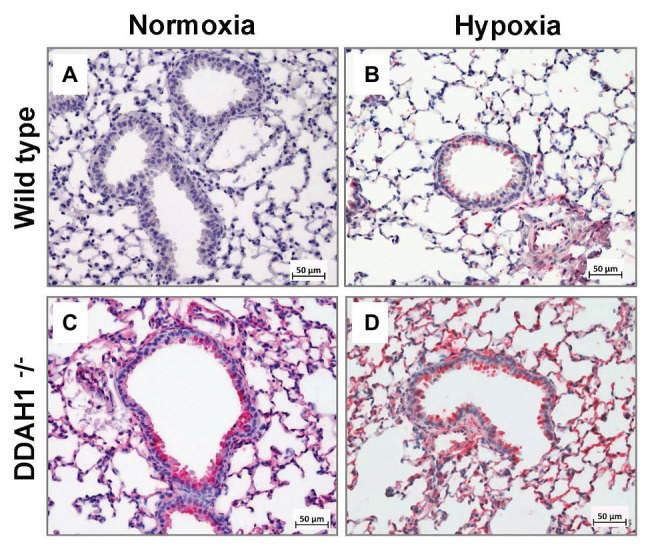
Immunohistochemical analysis of DDAH2 in mouse lungs. Representative lung sections stained for DDAH2 are shown from WT mice in normoxia **(A)**, WT mice in hypoxia **(B)**, DDAH1^−^/^−^ mice in normoxia **(C)**, and DDAH1^−^/^−^ mice in hypoxia **(D)**.

Immunohistochemical staining for DDAH2 confirmed the results of Western Blot analysis (Figure 5). Staining was most abundant in Ddah1^−^/^−^ mice after exposure to hypoxia and weakest in normoxic WT mice. DDAH2 was located mainly in bronchial epithelial cells and in type 2 alveolar epithelial cells within alveolae.

### Right Ventricular Systolic Pressure and Cardiomyocyte Hypertrophy

**Figure 6:**
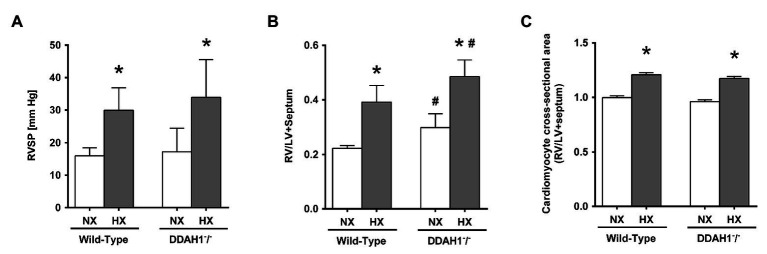
**(A)** Right ventricular systolic pressure (RVSP) in DDAH1^−^/^−^ mice and their wild-type littermates kept under normoxia or hypoxia for 4 weeks. **(B)** Right ventricular weight in relation to the weight of the left ventricle (LV) plus septum (Fulton index) in DDAH1^−^/^−^ mice and their wild-type littermates kept under normoxia or hypoxia for 4 weeks. **(C)** Cardiomyocyte cross-sectional area in DDAH1^−^/^−^ mice and their wild-type littermates kept under normoxia or hypoxia for 4 weeks. Cross-sectional area was expressed as ratio of cardiomyocyte area in the right ventricle (RV) over area of cardiomyocytes in the left ventricle and septum. At least 400 cardiomyocytes per heart were analyzed from at least four animals per group. All data are mean ± SEM of 8–12 animals per group. ^*^p < 0.05 vs. normoxia, ^#^*p* < 0.05 vs. wild-type. NX, normoxia (21% O_2_); HX, hypoxia (10% O_2_); LV, left ventricle; RV, right ventricle; RVSP, right ventricular systolic pressure.

Mean RVSP was not significantly different between Ddah1^−^/^−^ and WT mice under normoxic conditions. In hypoxia, RVSP significantly increased in both groups (WT, +88 ± 36%; Ddah1^−^/^−^, +97 ± 58%; both *p* < 0.05 vs. normoxia), with no significant difference between both genotypes (Figure 6A).

In Ddah1^−^/^−^ mice, we observed a slightly, but significantly higher right ventricular weight normalized to the weight of the left ventricle and septum in as compared to WT controls under normoxic conditions (0.30 ± 0.05 vs. 0.22 ± 0.02; *p* < 0.05). Hypoxia caused a significant and comparable increase of right ventricular weight by 76 ± 24% in the WT group and by 63 ± 19% in the Ddah1^−^/^−^ group (both *p* < 0.05 vs. normoxia; Figure 6B).

Immunohistological staining of the hearts revealed no significant difference of right ventricular cardiomyocyte cross-sectional area between WT and Ddah1^−^/^−^ mice under normoxic conditions. Hypoxia induced a significant increase in mean right ventricular cardiomyocyte cross-sectional area in both groups (Figure 6C).

## Discussion

The present study has three key findings: Firstly, chronic hypoxia causes a significant reduction of DDAH1 mRNA and protein expression and a significant increase in ADMA concentration in plasma and lung tissue in wild-type mice. The increase in plasma ADMA concentration in response to hypoxia is blunted in Ddah1^−^/^−^ mice that have virtually absent DDAH1 mRNA and protein expression and twice as high plasma ADMA concentration under normoxic conditions. Secondly, chronic hypoxia causes a significant increase in RVSP and right ventricular cardiomyocyte hypertrophy; the effect of hypoxia on these parameters was essentially identical in WT and Ddah1^−^/^−^ mice. Thirdly, DDAH2 protein expression increased in Ddah1^−^/^−^ mice in normoxia and was further elevated in hypoxia.

Pulmonary arterial hypertension in hypoxia is a consequence of sustained hypoxic pulmonary vasoconstriction, which occurs as a physiological response to low alveolar oxygen saturation (von Euler and Liljestrand, 1946). Numerous mechanisms have been discussed as potential mediators of this reaction of the pulmonary vasculature; and downregulation of eNOS or inhibition of its activity in the hypoxic lung has evolved as an important molecular component. ADMA is a competitive inhibitor of eNOS, resulting in a rapid and reversible inhibition of NO production under various pathophysiological conditions in the systemic circulation (for review, cf. Böger, 2006). Circulating ADMA is a marker of cardiovascular disease and mortality (Böger et al., 2009a), and mouse models in which DDAH – the major enzyme responsible for the metabolic degradation of ADMA – was genetically deleted show endothelial dysfunction and elevated systemic arterial pressure, while mice in which DDAH is overexpressed show decreased ADMA levels and protection from vascular damage (Leiper et al., 2007; Hu et al., 2011). Thus, ADMA and the pathways relating to its biosynthesis and metabolism are ideal candidates as molecular mechanisms contributing to hypoxic pulmonary vasoconstriction and high altitude pulmonary hypertension (HAPH).

Evidence has accumulated over the past years that the pathways of biosynthesis and metabolism of ADMA are present in the lungs and regulated by hypoxia: Protein arginine methyltransferases (PRMTs), which contribute to the *de novo* formation of ADMA, are highly expressed in lung tissue (Zakrzewicz and Eickelberg, 2009). Hypoxia was shown to upregulate PRMT2 expression and lung ADMA concentration (Yildirim et al., 2006). Both isoforms of DDAH (DDAH1 and DDAH2) are also present in the lungs. Although, we have not analyzed PRMT mRNA or protein expression in this study, the simultaneous elevation of ADMA and SDMA concentrations in lung tissue suggests that increased PRMT activity may have contributed to this effect.

We report here that in the WT mouse lung, mRNA and protein expression of DDAH1 is significantly reduced, while DDAH2 mRNA and protein expression is unaffected by hypoxia. This observation was paralleled by an about 43% higher circulating ADMA concentration and a doubling of ADMA concentration in lung tissue in hypoxia as compared to normoxia, by an elevation of RVSP, and an increase in right ventricular mass and cardiomyocyte hypertrophy in WT mice. All of these findings are in line with previous observations by Millatt and co-workers who demonstrated upregulation of eNOS protein but downregulation of DDAH1 protein levels, DDAH activity, ADMA concentration within lung tissue, and reduced NO generation after 1 week of normobaric hypoxia in rats (Millatt et al., 2003). After 30 days of chronic hypobaric hypoxia, we observed a significant elevation of tissue ADMA content, reduced DDAH activity and DDAH2 but not DDAH1 protein expression in the rat lung (Lüneburg et al., 2016). In that study, Ddah1 mRNA levels were unchanged, but Ddah2 mRNA levels were elevated in chronic hypoxia as compared to normoxic control conditions, and oxidative stress was increased. Thus, our previous findings point at possible destabilization of Ddah2 mRNA and/or at redox-mediated inactivation of DDAH catalytic activity in rats. DDAH1 was reported to be inactivated in the presence of oxidative or nitrosative stress (Lin et al., 2002; Knipp et al., 2003). In chronic hypoxia, oxidative stress may be derived from disruption of mitochondrial oxidation chain reactions (Freund-Michel et al., 2014) and/or from uncoupling of eNOS (Badran et al., 2016). These responses in the lung contrast with those reported for other organs: In the myocardium, repetitive ischemia increased DDAH1 protein expression (Zhang et al., 2017), and DDAH2 protein expression was increased in isolated peritoneal macrophages after exposure to hypoxia *in vitro* and in isolated peripheral blood monocytes from humans exposed to normobaric hypoxia *in vivo* (Lambden et al., 2016). However, hypoxia causes NO-dependent vasodilation in the organs within the systemic circulation, while hypoxic pulmonary vasoconstriction prevails in the lungs – the contrasting responses of enzymes regulating the NO-inhibitory molecule, ADMA, may thus fit well to the differences in hemodynamics between the pulmonary and systemic circulation in hypoxia.

In the Ddah1^−^/^−^ mouse lung, mRNA and protein expression of DDAH1 is virtually absent, while DDAH2 mRNA and protein expression is differentially regulated by hypoxia: Ddah2 mRNA remained unchanged in hypoxia vs. normoxia, while DDAH2 protein expression, which was elevated in normoxic Ddah1^−^/^−^ mice as compared to WT controls, further increased in hypoxia. These data suggest that DDAH2 may partially compensate for loss of DDAH1 in normoxic conditions, but in the presence of an additional stimulus like hypoxia, DDAH2 is further upregulated in mice lacking DDAH1. The upregulation of DDAH2 protein in hypoxia appears to be mediated by translational or post-translational processes rather than by transcriptional regulation, while the downregulation of DDAH1 in hypoxia seems to be brought about by downregulation of mRNA expression.

Our findings on differential regulation of DDAH1 and DDAH2 are in line with our observation that ADMA concentration did not increase in hypoxia beyond the levels measured in normoxic Ddah1^−^/^−^ mice, and that the impact of hypoxia on pulmonary vascular and right ventricular remodeling was not more pronounced in Ddah1^−^/^−^ mice than in WT littermates.

In the Ddah1^−^/^−^ mouse, ADMA concentrations in lung tissue and in plasma were elevated by about 50%, and a slight, but significant right ventricular hypertrophy was noted even under normoxic conditions. These findings are in line with impaired pulmonary arterial endothelium-dependent vasodilation and enhanced pulmonary arteriolar vasoconstriction that were shown under control conditions in a genetically different, heterozygous Ddah1^−^/^+^ mouse model (Leiper et al., 2007). However, the effects of chronic hypoxia on RVSP and right ventricular hypertrophy were not significantly different between WT and Ddah1^−^/^−^ mice in our study, which corresponds to the absence of a significant difference in lung tissue ADMA concentration between both genotypes in hypoxia. These findings suggest that upregulation of DDAH2 protein may limit a further increase in ADMA and its subsequent pathophysiological consequences. Although it has been debated whether DDAH2 is enzymatically active toward ADMA (Hu et al., 2011), siRNA-mediated knockdown of DDAH2 impaired NO-mediated vascular function (Wang et al., 2007). The latter observation is in line with our hypothesis presented here. In combination with previous findings by others, these data support the view that acute hypoxic pulmonary vasoconstriction and chronic hypoxic pulmonary hypertension and right ventricular hypertrophy are differentially affected by the various components of ADMA biosynthesis and metabolism. Both available mouse models of genetic Ddah1 deletion show a consistent pattern of inhibition of DDAH activity vs. elevation of circulating ADMA concentration: In the model generated by Leiper and co-workers, heterozygous knockout of Ddah1 resulted in about 50% reduction of total DDAH activity, which was associated with an about 21% increase in plasma ADMA concentration (Leiper et al., 2007). In our mouse model, homozygous knockout of Ddah1 resulted in about 95% loss of total DDAH activity, which was associated with an about 130% increase in plasma ADMA concentration as compared to WT littermates (Hu et al., 2011), an observation that is reproduced in the present study [difference in ADMA plasma concentration between WT and Ddah1^−^/^−^, +93.2 ± 10.3% (day 1), +139.6 ± 14.1% (day 28) in normoxia]. By contrast, hypoxic dysregulation of DDAH1, which caused a small, 37% decrease in DDAH1 protein in the rat lungs, was consistently associated with a 37% reduction in total lung DDAH activity, but a huge, 2.3-fold increase in pulmonary ADMA concentration (Millatt et al., 2003). This finding suggests that other enzymes involved in the regulation of ADMA may also be regulated by hypoxia. In line with this observation, chronic hypoxia caused upregulation of PRMTs and elevation of lung ADMA content in another study (Yildirim et al., 2006), and both, ADMA and SDMA concentration in lung tissue were elevated in this study. Moreover, DDAH1 overexpression (which causes an increase in total DDAH activity) decreased sustained hypoxic pulmonary vasoconstriction but it did not modulate long-term HAPH (Bakr et al., 2013). Finally, normobaric hypoxia increased DDAH2 protein by 4.5-fold, reduced ADMA concentration by 24%, and increased NO production in monocytes (Lambden et al., 2016). Taken together, these findings suggest that ADMA is involved in the response of the pulmonary circulation to hypoxia, but various enzymes may differentially regulate its concentration in the different cell types present in the lung.

Our study has several limitations. Firstly, we were unable to analyze all genes and enzymes involved in the complex regulation of dimethylarginine biosynthesis and metabolism. Rather, the focus of our study was to dissect the differential roles of the two isoforms of DDAH in the response to hypoxia. Furthermore, we did not analyze NO production in lung tissue or systemically, as this would have required intervention with stable isotope-labeled L-arginine, and we therefore cannot conclusively prove that inhibition of NO production is the sole mechanism of action of the changes in DDAH expression and in ADMA concentration. However, our initial, detailed description of the Ddah1^−^/^−^ mouse model used here (Hu et al., 2011) as well as previous publications by our group in which we have repeatedly demonstrated in different *in vitro* and animal models that ADMA inhibits the conversion of L-[^15^N_2_]-arginine to ^15^NO by NO synthase (NOS) provide plenty of evidence that ADMA inhibits NOS catalytic activity (Böger et al., 2000, 2004). It may therefore be justified to conclude that NOS inhibition is the main mechanism of action of the pathophysiological changes that we observed, because no other biological activity for ADMA has been demonstrated to date.

In summary, our present data support the view that modulation of the NO pathway by ADMA is an important mechanism of hypoxia-induced changes in the lung circulation. However, ADMA is regulated by multiple enzymatic pathways, which are differentially modulated in hypoxia. In WT mice, downregulation of DDAH1 may be the major cause for elevation of ADMA in hypoxia, while in Ddah1^−^/^−^ mice, DDAH2 protein is upregulated, limiting further dysregulation of ADMA-related pathways and, by this, pulmonary hypertension and cardiac hypertrophy in hypoxia.

## Data Availability Statement

The raw data supporting the conclusions of this article will be made available by the authors, without undue reservation.

## Ethics Statement

The animal study was reviewed and approved by Amt für Gesundheit und Verbraucherschutz Hamburg.

## Author Contributions

JHa, NL, LH, and RB designed the study. JHa, AG, JHi, JZ, US, NL, and LH were involved in data acquisition. Data analysis and interpretation was performed by JHa, AG, and RB. JHa drafted the manuscript. All other authors critically reviewed the manuscript. RB supervised the study. Funding for the study was obtained by JHa, NL, LH, and RB. All authors contributed to the article and approved the submitted version.

### Conflict of Interest

The authors declare that the research was conducted in the absence of any commercial or financial relationships that could be construed as a potential conflict of interest.
